# Genome-wide prediction and analysis of human tissue-selective genes using microarray expression data

**DOI:** 10.1186/1755-8794-6-S1-S10

**Published:** 2013-01-23

**Authors:** Shaolei Teng, Jack Y Yang, Liangjiang Wang

**Affiliations:** 1 Department of Genetics and Biochemistry, Clemson University, Clemson, SC 29634, USA; 2 Harvard Medical School, Harvard University, P.O. Box 400888, Cambridge, MA 02115, USA; 3 J.C. Self Research Institute of Human Genetics, Greenwood Genetic Center, Greenwood, SC 29646, USA

## Abstract

**Background:**

Understanding how genes are expressed specifically in particular tissues is a fundamental question in developmental biology. Many tissue-specific genes are involved in the pathogenesis of complex human diseases. However, experimental identification of tissue-specific genes is time consuming and difficult. The accurate predictions of tissue-specific gene targets could provide useful information for biomarker development and drug target identification.

**Results:**

In this study, we have developed a machine learning approach for predicting the human tissue-specific genes using microarray expression data. The lists of known tissue-specific genes for different tissues were collected from UniProt database, and the expression data retrieved from the previously compiled dataset according to the lists were used for input vector encoding. Random Forests (RFs) and Support Vector Machines (SVMs) were used to construct accurate classifiers. The RF classifiers were found to outperform SVM models for tissue-specific gene prediction. The results suggest that the candidate genes for brain or liver specific expression can provide valuable information for further experimental studies. Our approach was also applied for identifying tissue-selective gene targets for different types of tissues.

**Conclusions:**

A machine learning approach has been developed for accurately identifying the candidate genes for tissue specific/selective expression. The approach provides an efficient way to select some interesting genes for developing new biomedical markers and improve our knowledge of tissue-specific expression.

## Background

Understanding how different tissues achieve specificity is a fundamental question in tissue ontogenesis and evolution. Some genes are highly expressed in a particular tissue and lowly expressed or not expressed in other tissues. These genes are generally called tissue-selective genes. The genes are responsible for specialized functions in particular tissues, and thus can serve as the biomarkers for specific biological processes. In addition, many tissue-selective genes are involved in the pathogenesis of complex human diseases [1], including insulin signaling pathways in diabetes [2] and tumor-host interactions in cancer [3]. Since the majority of disease genes have the tendency to be expressed preferentially in particular tissues [4], identifying tissue-selective genes is also important for drug target selection in biomedical research. Tissue-specific genes, which are specifically expressed in a particular tissue, are regarded as the special case of tissue selective genes. The identification of tissue-specific genes could help biologists to elucidate the molecular mechanisms of tissue development and provide valuable information for identifying candidate biomarkers and drug targets.

Different methods have been used to identify and characterize tissue-specific genes. Traditional experimental methods, including RT-PCR and Northern blot, are usually carried out at the single-gene level and thus time-consuming. High-throughput technologies, such as Expressed Sequence Tag (EST) sequencing and DNA microarrays, have the capacity to perform genome-wide analysis with high efficiency. The DNA microarray technology can generate large amounts of gene expression data from various tissues, and provide the useful data source for analyzing tissue-specific genes. Several statistical methods have been applied for identifying tissue-specific genes using gene expression data. Kadota and co-workers [5] described an unsupervised method to select the tissue-specific genes using Akaike's information criterion (AIC) approach. Another method called ROKU [6] has been developed by the same group for detecting tissue-specific gene expression patterns. The approach used Shannon entropy and outlier detection to scan expression profiles for ranking tissue-specific genes. Liang *et al*. [7] developed a statistical method based on hypothesis testing procedures to profile and identify the tissue-selective genes. However, the statistical methods for tissue-specific gene prediction suffer from drawbacks. The microarray expression data are generated from different experiments, both biological variations and experimental noise result in significant variations in data quality. The statistical methods usually assigned an equal weight to each observation for prediction. Thus, the methods do not work well for non-linear models and may not detect the hidden expression patterns from the noisy microarray data. Moreover, the statistical methods do not use biological knowledge for prediction. The simple data-driven analysis may produce some misleading results for further experimental studies.

Machine learning can automatically recognize hidden patterns in complex data. It has been shown that machine learning can be used to construct accurate classifiers for tissue-specific gene prediction. Chikina *et al*. [8] used Support Vector Machines (SVMs) to predict tissue-specific gene expression in *Caenorhabditis elegans *with whole-animal microarray data. The SVM classifiers reached high predictive performances in nearly all tissues. It was shown that the approach outperformed clustering methods and provided valuable information for further experimental studies. However, it is still unknown whether machine learning methods can be used to predict tissue-specific genes in human.

We previously compiled a large microarray gene expression dataset, which contained 2,968 expression profiles of various human tissues, including brain, liver, testis, blood and kidney samples [9]. A computational method was also developed for identifying tissue-selective genes using the integrated microarray dataset. However, the method assigned an equal weight to each expression profile for identification. In this study, a machine learning approach was developed for human tissue-specific/selective gene prediction using the available dataset. According to the lists of known tissue-specific/selective genes, the gene expression data were extracted from the compiled dataset and used for classifier construction. Random Forests (RFs) and Support Vector Machines (SVMs) were trained with the expression data to construct accurate classifiers. The results indicate that the RF classifiers achieved better predictive performance for tissue-specific gene prediction. The approach generated large numbers of candidate genes for brain and liver-specific expression. The examinations of high scoring genes suggest that our approach can be used to select candidate genes for experimental studies.

## Methods

**Figure 1:**
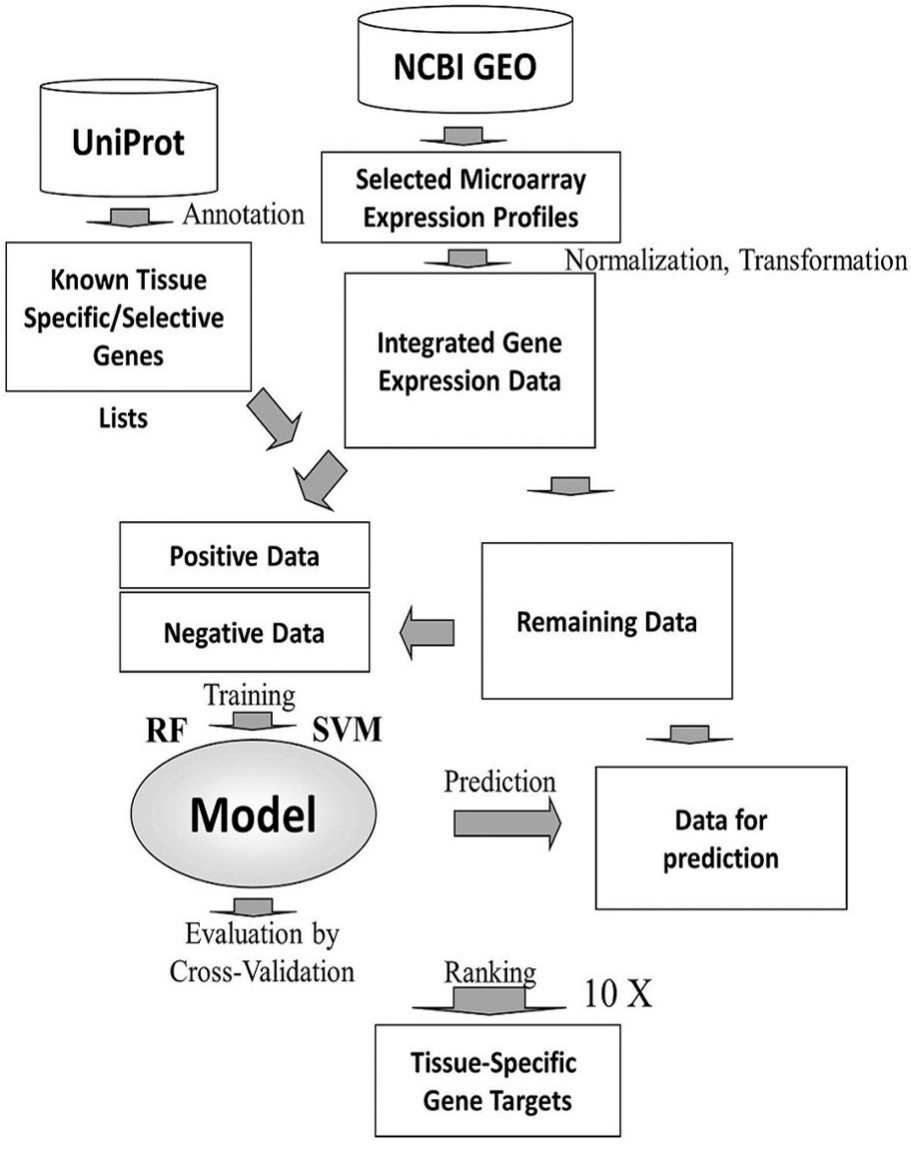
**Schematic diagram of the approach for predicting tissue-specific genes**.

A schematic diagram of the approach used in this study is shown in Figure 1. The microarray expression profiles of various human tissues were compiled from previous studies, and integrated into a single dataset through normalization and transformation. The lists of known tissue-specific genes were manually collected from UniProt database. The tissue-specific gene expression data were extracted from the integrated single dataset and labelled as positive training instances. The remaining expression data were randomly divided into two subsets. The negative dataset contained tenfold number of data instances as the positive instances. Random Forests (RFs) and Support Vector Machines (SVMs) were trained with the training instances to construct classifiers. The tenfold cross-validation method was performed to evaluate the classifier performance. The models were then used to score the remaining data instances for prediction. The classifier construction and prediction were repeated ten times, and the candidate genes were prioritized according to their average classifier outputs from ten predictions.

### Microarray data collection and integration

The approach for compiling human microarray expression profiles was described in our previous study [9]. The microarray gene expression profiles from 131 microarray studies (different experimental batches) were collected from the NCBI GEO database. These expression profiles were generated using the Affymetrix HG-U133 Plus 2.0 Array with 54,613 probe sets. The statistical model-based method, dChip [10], was used for microarray data normalization. The raw data in CEL file format were divided into different normalization groups. The invariant set method [11] was used to normalize each group of microarray profiles to minimize the batch effect (variation across different microarray studies). After normalization, global median transformation was used to integrate the microarray profiles into a single dataset. The dataset used in this study contained 2,968 expression profiles of various human tissues, including brain (616 profiles), liver (117 profiles), testis (36 profiles), blood (409 profiles) and kidney (73 profiles).

### Training data preparation

Tissue-selective genes are defined as the genes whose expression is enriched for one or a few similar tissue types. The genes were manually collected from the UniProt database. The particular tissue name was used as a query and the reviewed human genes were selected for preparation. The tissue-selective genes are defined as the genes that are preferentially expressed in a particular tissue from the descriptions of their annotations. Most of the genes are identified by the experimental methods, which are independent from the microarray expression data in the list. In this study, 408 brain-selective genes, 96 liver-selective genes, 326 testis-selective genes, 324 blood-selective genes and 45 kidney-selective genes were collected from UniProt database. Tissue-specific genes, whose expression is specific to only one particular tissue type, are considered as the special case of tissue-selective genes. 289 brain-specific genes and 69 liver-specific genes were selected from the corresponding tissue-selective genes with the annotation that their expression is specific to only brain or liver. Tissue specific-genes can be used to construct the highly accurate classifiers. Thus, the tissue specific-gene prediction is the focus of the present study.

According to the known tissue-specific/selective gene lists, the tissue-specific/selective gene expression data was retrieved from the integrated microarray dataset and labelled as the positive training instances. The probe sets with detectable expression signals in corresponding tissue samples were selected for classifier construction. For tissue-specific gene prediction, the expression values for 403 probe sets of brain-specific genes and 90 probe sets of liver-specific genes were used for input vector encoding. 692 probe sets of brain-selective genes, 150 probe sets of liver-selective, 430 probe sets of testis-selective genes, 456 probe sets of blood-selective genes and 76 probe sets of kidney-selective genes were used for tissue-selective gene prediction.

The negative examples were defined as the genes that do not have preferential expression in particular tissues. For this study, we randomly selected the data instances from the remaining data and labelled as the negative training instances. The number of negative instances was set as tenfold with positive instances to make enough data instances for training. The negative and positive data instances were combined as the training dataset to construct classifiers using machine learning algorithms. The remaining probes were used as the candidate genes for prediction with the classifiers constructed from the training dataset.

### Random Forests

The use of 2,968 expression profiles for input vector encoding gives the same number of input variables. One potential problem is model overfitting since there were only a small number of positive instances (probe sets of known tissue-specific genes) available for this study. We thus used the Random Forest (RF) learning algorithm, which could handle a large number of input variables and avoid model overfitting through random feature selection. The randomForest package in R [12] was used in this study for classifier construction. The number of trees in a classifier (*ntree*) and the number of variables selected to split each node (*mtry*) were set to 1000 and 6, respectively. The classifier performance did not show significant improvement by using other values for the *mtry *and *ntree *parameters.

### Support Vector Machines

Support Vector Machines (SVMs) are widely used for binary classification [13]. In this study, SVM classifiers were constructed and compared with RF classifiers for identifying human tissue-specific genes. The SVMlight software package (http://svmlight.joachims.org/) was used to construct the SVM classifiers with the linear kernel function [14]. The polynomial and radial basis function (RBF) kernels were also tested for classifier construction, but the classifiers did not achieve high predictive performances in cross-validation tests.

### Classifier evaluation and prediction

In this study, a tenfold cross-validation approach was used to evaluate classifiers with the following performance measures:

(1) $$\text{Accuracy (AC)}=\frac{TP+TN}{TP+TN+FP+FN}$$

(2) $$\text{Sensitivity (SN)}=\frac{TP}{TP+FN}$$

(3) $$\text{Specificiy (SP)}=\frac{TN}{TN+FP}$$

(4) $$\text{MCC}=\frac{TP\times TN-FP\times FN}{\sqrt{\left(TP+FP\right)\left(TP+FN\right)\left(TN+FP\right)\left(TN+FN\right)}}$$

where TP is the number of true positives; TN is the number of true negatives; FP is the number of false positives; and FN is the number of false negatives. The Receiver Operating Characteristic (ROC) curve [15] and the area under the curve (AUC) [16] were also used for classifier evaluation and comparison.

The classifier construction and prediction were repeated ten times. In each run, classifier performance was evaluated using the above measures. The classifier was then used to predict tissue-specific genes in the human genome. The tissue-specific gene targets were sorted according to the average value of classifier outputs from ten predictions, and a higher value might indicate a higher probability of being expressed predominantly in a particular tissue.

### Go enrichment and promoter sequence analyses

DAVID (http://david.abcc.ncifcrf.gov/), a web-based tool for go enrichment analysis, was used for integrating functional annotations of predicted tissue-specific genes [17]. The probe names of candidate targets were used as the inputs, and "GOTERM_BP_FAT", "GOTERM_CC_FAT" and "GOTERM_MF_FAT" were utilized as the Gene Ontology search option.

The 500 bp upstream sequences of predicted tissue-specific genes were downloaded from the UCSC Table Browser (http://genome.ucsc.edu/cgi-bin/hgTables) with the gene names of candidate targets as the inputs. The promoter sequences were used to identify the regulatory DNA motifs with SCOPE web server (http://genie.dartmouth.edu/scope/). SCOPE [18] applied three learning algorithms to find different type of sequence motifs, including non-degenerate motifs, degenerate motifs, and bipartite motifs. The candidate regulatory motifs used in this study are the high scoring motifs (motif width ≥ 6) returned from the combination of the prediction results. DiRE [19] (http://dire.dcode.org/) were used to analyse the candidate transcription factors of predicted tissue-specific genes. The gene names of candidate targets were used as the inputs, and the transcription factors for the regulatory elements of candidate targets identified with the Enhancer Identification method were reported.

## Results and discussion

### Dataset validation

**Figure 2:**
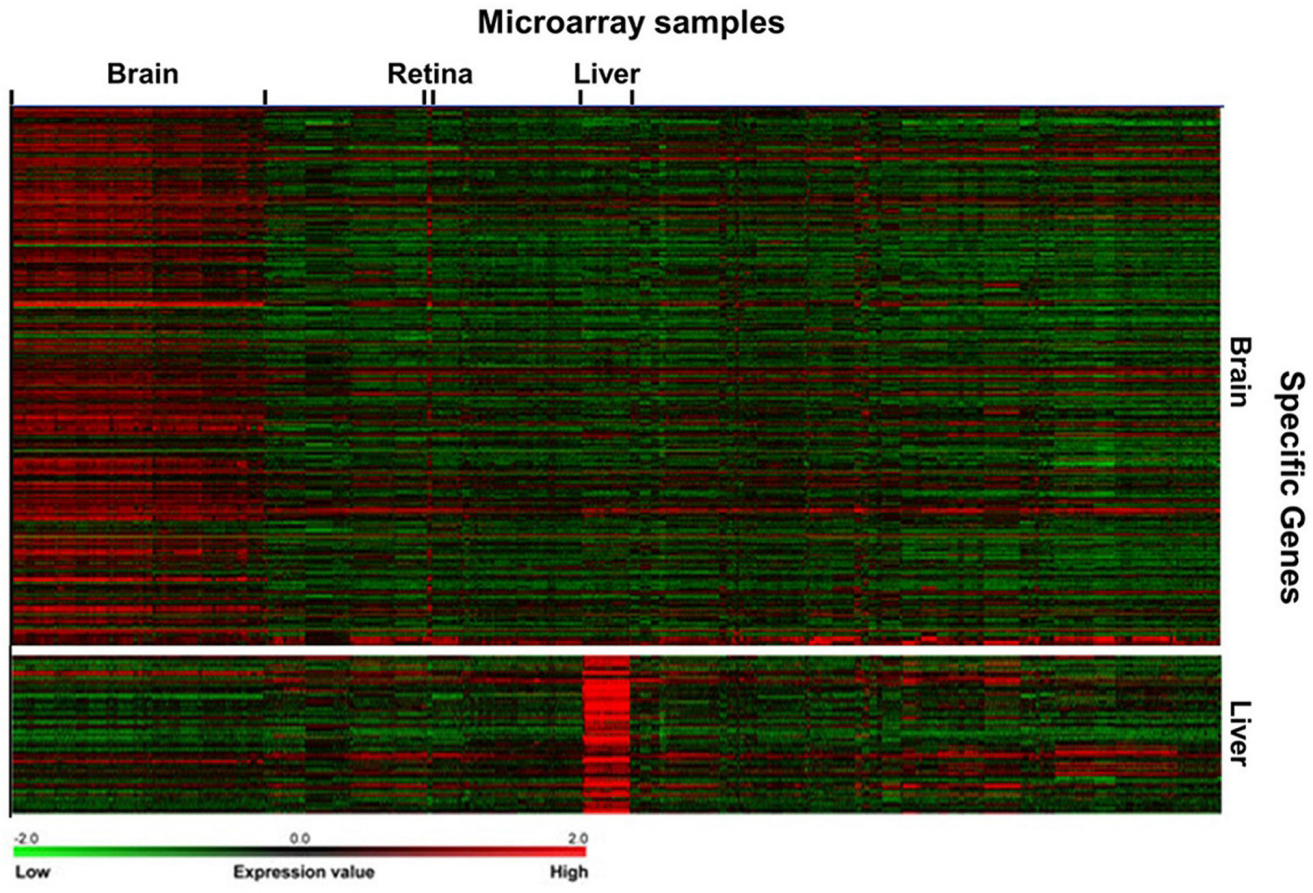
**Visualization of known tissue-specific gene expression patterns**.

The known tissue-specific genes are expressed predominantly in particular tissues, so the transcripts of the genes were expected to be detected in corresponding tissue samples in the integrated microarray dataset. To visualize the expression patterns of the known tissue-specific genes, TM4 MeV [20] was used to generate the heat maps for brain and liver-specific genes. As shown in Figure 2, the known brain-specific genes have expression patterns in brain as well as retina samples. Since retina shares a common embryonic origin with brain and translates visual images into nerve signals for brain, retina is considered as the sensory part of the brain. Thus, the known brain-specific genes may also have some expression levels in retina samples.

The transcripts of known liver-specific genes are detected clearly in liver samples (Figure 2). The results suggest that the expression data according to our lists of known tissue-specific genes can provide useful information for classifier construction using machine learning methods. It is noteworthy that some probe sets of known tissue-specific genes have high expression or no expression for all tissue samples. To improve the quality of classifiers, the probes without detectable expression signals in all the samples are excluded from the training dataset.

### Prediction of tissue-specific genes

**Figure 3:**
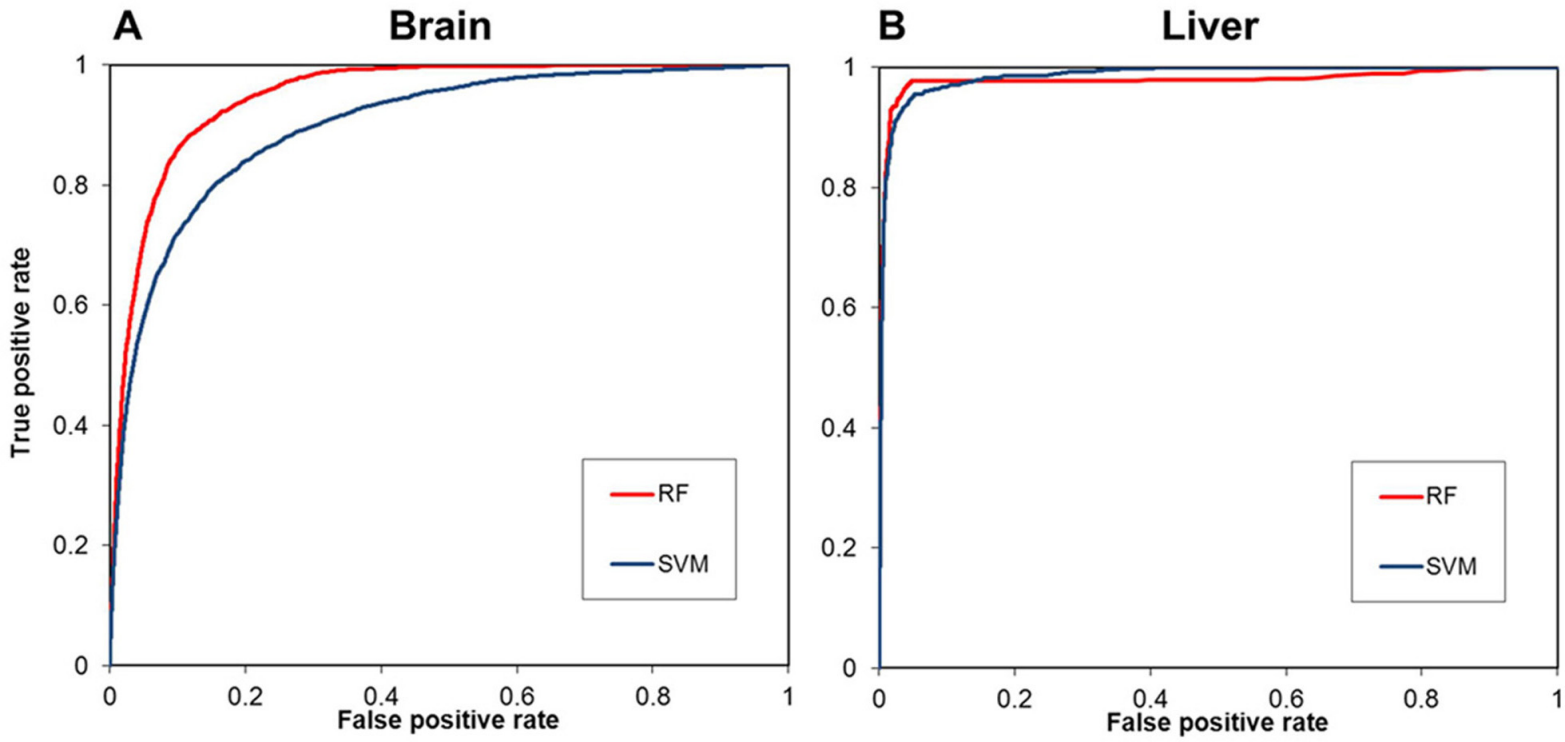
**ROC curves to compare the performances of RF and SVM classifiers for predicting tissue-specific genes**.

**Figure 4:**
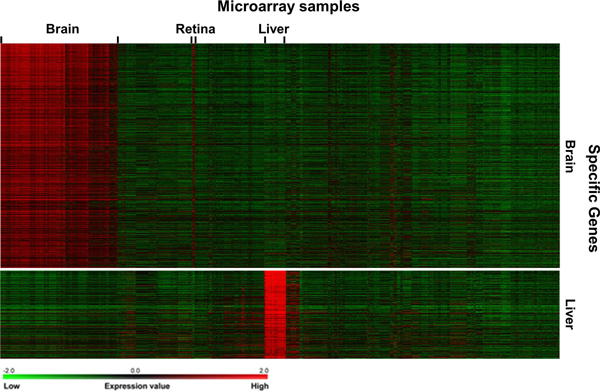
**Visualization of predicted tissue-specific gene expression patterns**.

**Figure 5:**
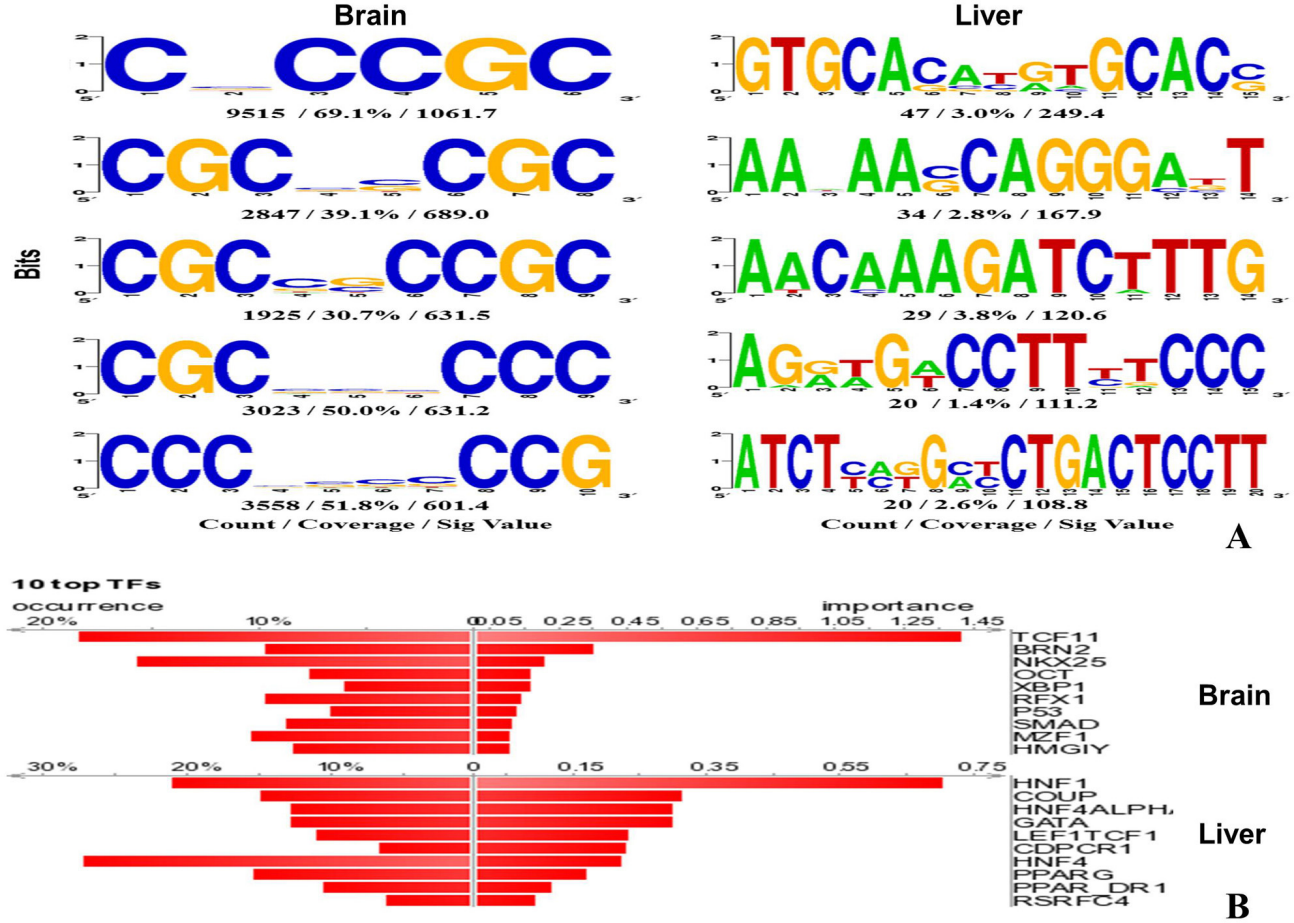
**Promoter sequence analysis of predicted tissue-specific genes**. (**A**) Regulatory DNA motif over-represented in the promoter sequence of candidate targets. (**B**) Candidate transcription factors (TFs) of predicted tissue-specific genes. Figures are generated using SCOPE (http://genie.dartmouth.edu/scope/) and DiRE (http://dire.dcode.org/), respectively.

Random Forests (RFs) and Support Vector Machines (SVMs) were used to construct classifiers for predicting brain and liver-tissue specific genes. The results suggest that RF classifiers reached better predictive performance than SVM models (Table 1 and Figure 3). We identified 1,408 brain-specific microarray probes (1,126 genes) and 493 liver-specific probes (357 genes) using RF classifiers (Additional file 1 and 2), which are more than the tissue-selective genes identified in the previous study (222 brain-selective genes and 69 liver-selective genes) [9]. High scoring gene targets with brain or liver-specific expression have been examined (Tables 2 and 3), and the results suggest that the approach can be used to identify new gene targets for biomedical research. Moreover, it was shown that the transcripts of candidate genes could be detected clearly in corresponding tissue samples (Figure 4), and the functions of the predicted targets were consistent with tissue origins in GO enrichment analysis (Tables 4 and 5). The regulatory DNA motifs were identified based on the promoter sequences of the predicted tissue-specific genes, and the candidate transcription factors were previously shown to regulate the specialized functions in particular tissues (Figure 5).

**Table 1:** Comparison of Random Forest and Support Vector Machine classifiers for predicting tissue-specific genes.

Tissue	Method	AC (%)	SN (%)	SP (%)	MCC	ROC AUC
Brain	SVM	92.07 (± 0.302)	54.23 (± 1.227)	95.82 (± 0.263)	0.5091 (± 0.015)	0.8937 (± 0.003)
	
	RF	93.48 (± 0.240)	53.73 (± 1.485)	97.43 (± 0.153)	0.5676 (± 0.016)	0.9488 (± 0.002)

Liver	SVM	97.29 (± 0.421)	84.11 (± 2.281)	98.61 (± 0.309)	0.8350 (± 0.025)	0.9854 (± 0.004)
	
	RF	97.29 (± 0.341)	79.00 (± 1.355)	99.12 (± 0.255)	0.8290 (± 0.0213)	0.9777 (± 0.002)

The values outside and inside brackets are the average value and standard deviation of measures in ten classifier evaluations, respectively.

**Table 2:** List of high-scoring genes with specific expression in the brain.

Probe	Gene	Description	Score*
223654_s_at	BRUNOL4	Bruno-like 4, RNA binding protein (Drosophila)	0.8753
227440_at	ANKS1B	Ankyrin repeat and sterile alpha motif domain containing 1B	0.8685
230280_at	TRIM9	Tripartite motif-containing 9	0.866
238966_at	BRUNOL4	Bruno-like 4, RNA binding protein (Drosophila)	0.8345
205143_at	NCAN	Neurocan	0.832
204762_s_at	GNAO1	Guanine nucleotide binding protein (G protein), alpha activating activity polypeptide O	0.8201
232276_at	HS6ST3	Heparan sulfate 6-O-sulfotransferase 3	0.8186
203619_s_at	FAIM2	Fas apoptotic inhibitory molecule 2	0.8124
241998_at	LOC389073	Similar to RIKEN cDNA D630023F18	0.8074
206381_at	SCN2A	Sodium channel, voltage-gated, type II, alpha subunit	0.8021
203069_at	SV2A	Synaptic vesicle glycoprotein 2A	0.7998
1557256_a_at	AA879409	CDNA FLJ37672 fis, clone BRHIP2012059	0.797
229039_at	SYN2	Synapsin II	0.7956
242651_at	AI186173	Transcribed locus	0.7951
227453_at	UNC13A	unc-13 homolog A (C. elegans)	0.7888
203618_at	FAIM2	Fas apoptotic inhibitory molecule 2	0.7744
229463_at	NTRK2	Neurotrophic tyrosine kinase, receptor, type 2	0.7728
214111_at	OPCML	Opioid binding protein/cell adhesion molecule-like	0.7722
214376_at	AI263044	Clone 24626 mRNA sequence	0.7668
220131_at	FXYD7	FXYD domain containing ion transport regulator 7	0.7662

* Score: the average value of RF classifier outputs from ten predictions.

**Table 3:** List of high-scoring genes with specific expression in the liver.

Probe	Gene	Description	Score*
206610_s_at	F11	Coagulation factor XI (plasma thromboplastin antecedent)	0.7869
1554491_a_at	SERPINC1	Serpin peptidase inhibitor, clade C member 1	0.7737
219465_at	APOA2	Apolipoprotein A-II	0.7609
217512_at	BG398937	Unknown	0.7559
207102_at	AKR1D1	Aldo-keto reductase family 1, member D1	0.7466
207218_at	F9	Coagulation factor IX	0.725
210168_at	C6	Complement component 6	0.7239
204987_at	ITIH2	Inter-alpha (globulin) inhibitor H2	0.7191
209978_s_at	LPA/PLG	Lipoprotein, Lp(a)/plasminogen	0.7191
214069_at	ACSM2	Acyl-CoA synthetase medium-chain family member 2	0.7099
206345_s_at	PON1	Paraoxonase 1	0.7004
206651_s_at	CPB2	Carboxypeptidase B2 (plasma)	0.6959
241914_s_at	ACSM2	Acyl-CoA synthetase medium-chain family member 2	0.6945
206840_at	AFM	Afamin	0.6846
206410_at	NR0B2	Nuclear receptor subfamily 0, group B, member 2	0.6837
214842_s_at	ALB	Albumin	0.6809
217319_x_at	CYP4A11	Cytochrome P450, family 4, subfamily A, polypeptide 11	0.6772
242817_at	PGLYRP2	Peptidoglycan recognition protein 2	0.6765
207407_x_at	CYP4A11	Cytochrome P450, family 4, subfamily A, polypeptide 11	0.6752
231398_at	SLC22A7	Solute carrier family 22, member 7	0.6746

* Score: the average value of RF classifier outputs from ten predictions.

**Table 4:** GO term enrichment analysis of predicted brain-specific genes.

Category	Term	Count*	%*	P-Value*
GOTERM_CC_FAT	GO:0045202~synapse	103	11.41	1.37E-49
GOTERM_CC_FAT	GO:0044456~synapse part	83	9.19	2.68E-45
GOTERM_BP_FAT	GO:0019226~transmission of nerve impulse	80	8.86	5.25E-36
GOTERM_CC_FAT	GO:0043005~neuron projection	85	9.41	4.00E-35
GOTERM_BP_FAT	GO:0007268~synaptic transmission	73	8.08	6.82E-35
GOTERM_MF_FAT	GO:0005216~ion channel activity	76	8.42	2.29E-30
GOTERM_MF_FAT	GO:0022838~substrate specific channel activity	77	8.53	3.03E-30
GOTERM_MF_FAT	GO:0022836~gated channel activity	68	7.53	4.80E-30
GOTERM_MF_FAT	GO:0015267~channel activity	77	8.53	3.28E-29
GOTERM_MF_FAT	GO:0022803~passive transmembrane transporter activity	77	8.53	3.87E-29

*Count: the number of genes involved in the given GO term; %: the percentage of involved genes in total genes; P-Value: the modified Fisher Exact P-Value.

**Table 5:** GO term enrichment analysis of predicted liver-specific genes.

Category	Term	Count*	%*	P-Value*
GOTERM_BP_FAT	GO:0002526~acute inflammatory response	29	8.41	1.65E-24
GOTERM_BP_FAT	GO:0009611~response to wounding	55	15.94	8.55E-23
GOTERM_CC_FAT	GO:0005615~extracellular space	63	18.26	8.65E-23
GOTERM_CC_FAT	GO:0005576~extracellular region	109	31.59	1.35E-21
GOTERM_BP_FAT	GO:0007596~blood coagulation	25	7.25	5.55E-19
GOTERM_BP_FAT	GO:0050817~coagulation	25	7.25	5.55E-19
GOTERM_BP_FAT	GO:0007599~hemostasis	25	7.25	2.33E-18
GOTERM_BP_FAT	GO:0055114~oxidation reduction	54	15.65	2.46E-18
GOTERM_BP_FAT	GO:0006956~complement activation	18	5.22	2.70E-18
GOTERM_BP_FAT	GO:0002541~activation of plasma proteins involved in acute inflammatory response	18	5.22	4.37E-18

*Count: the number of genes involved in the given GO term; %: the percentage of involved genes in total genes; P-Value: the modified Fisher Exact P-Value.

In this study, we constructed both RF and SVM classifiers for predicting brain and liver-specific genes. 403 probe sets of brain-specific genes and 90 probe sets of liver-specific genes were used for classifier construction. For brain-specific gene prediction, the RF classifier achieved the AUC value at 0.9488 (Table 1), which is significantly higher than the AUC value of SVM classifier (AUC = 0.8937). The RF classifier reached 53.73% sensitivity and 97.43% specificity, and MCC = 0.5676. For liver-specific gene prediction, the SVM classifier gave MCC = 0.8350 and ROC AUC = 0.9854. The RF classifier achieved a similar level of performance with MCC = 0.8290 and ROC AUC = 0.9777. Thus, the results suggest that the RF algorithm performs better for predicting tissue-specific genes in this study.

The ROC curves of RF and SVM classifiers for predicting brain-specific genes and live-specific genes have been compared in Figure 2. The ROC curves of RF and SVM classifiers are not significantly different for the prediction of liver-specific genes (Figure 2b). However, The ROC curve of RF classifier was clearly better than the SVM classifier for the prediction of brain-specific genes (Figure 2a). The results confirm that RF classifier outperforms the SVM models for tissue-specific gene prediction. The possible reason is that RFs can handle a large number of input variables and avoid model overfitting. The use of 2,968 expression profiles for input vector encoding results in the same large number of input variables, which may lead to model overfitting. Interestingly, the RF algorithm can handle the situation and show better predictive performance in the present study.

### Brain-specific gene expression

The human brain gives us the ability to think and sets us apart from other animals. It has a highly complex structure which contains different regions with specific functions. For example, the hippocampus is involved in spatial navigation and long-term memories, whereas the cerebral cortex plays key roles in language, attention and consciousness. Any damage in these regions results in various mental disorders including Alzheimer's disease, Parkinson's disease and Mood disorder. In this study, the predicted brain-specific genes are expected to have preferential expression in the brain, and may play important roles in neuron functions such as synaptic transmission and neuronal migration.

In the study, 1,408 candidate targets with positive scores (the average value of classifier outputs from ten predictions) were predicted as the brain-specific genes (Additional file 1). In Figure 4, the expression patterns of candidate gene targets using RF classifier are visualized with the heat maps generated using TM4 MeV. The predicted targets show clear expression in brain samples, which indicates that our approach is useful for brain-specific gene prediction. Similar to the known brain-specific genes, the transcripts of the predicted targets are also detected in retina samples. GO enrichment analysis of the candidate targets demonstrates that many candidate genes have basic neuron functions (Table 4). For example, neurotransmission is an electrical or chemical signal motion within synapses caused by transmission of a nerve impulse. The predictions are enriched for neurotransmission-related GO terms such as "synapse", "synapse part", "transmission of nerve impulse", "neuron projection", "synaptic transmission" and "passive transmembrane transporter activity". Some channel-related GO terms including "ion channel activity", "substrate specific channel activity", "gated channel activity" and "channel activity" are detected in the enrichment analysis of our predictions.

To understand the regulatory signals of human tissue-specific genes, SCOPE [18] was used to search for the overrepresented DNA motifs in the promoters of the predicted brain-specific genes (Figure 5a). Some transcription factors might bind to the DNA motifs in the promoter regions and regulate the tissue-specific gene expression. DiRE [19] was used to identify the candidate transcription factors of the predicted brain-specific genes (Figure 5b). Interestingly, the identified transcription factors might be involved in regulating neuron development and function. For example, *BRN2 *and *OCT *belong to the POU domain protein family, and are involved in the differentiation and function of neural cells [21,22]. X-box-binding protein 1 (*XBP1*) is activated in neural development and plays a key role for the unfolded protein response in the endoplasmic reticulum [23]. Regulatory factor X1 (*RFX1*) can enhance the expression of excitatory amino acid transporters (*EAAT*) to regulate the glutamate neurotransmission [24]. The *p53 *protein is important for various cellular processes, including apoptosis, differentiation, DNA repair and cell-cycle arrest, in neurons [25]. The results suggest that the approach developed in the present study can provide valuable information for studying the regulatory modules of tissue-specific genes.

The predicted targets have not been annotated as brain-specific genes in the UniProt database. However, recent studies suggest that some of the predicted targets, including *BRUNOL4, ANKS1B, TRIM9, NCAN, FAIM2, OPCML *and *FXYD7 *(Table 2), are expressed predominantly in the brain. For example, the RNA-binding protein encoded by *BRUNOL4 *plays an important role in many cellular processes including RNA stability, pre-mRNA alternative splicing, mRNA editing and translation [26,27]. It was shown that the protein was predominantly expressed in the brain with enrichment in the hippocampus [28]. In this study, the probes of *BRUNOL4 *have the highest (223654_s_at, 0.8753) and fourth-ranked (238966_at, 0.8600) scores. *ANKS1B *encodes Amyloid-beta protein which can regulate the nucleoplasmic coilin protein interactions in neuronal cells. Previous studies showed that the protein is mainly expressed in brain and may be implicated in Alzheimer's disease [29]. Brain-specific E3 ligase encoded by *TRIM9 *has a high level of expression in the cerebral cortex and may be involved in the pathogenesis of Parkinson's disease [30]. Neurocan (*NCAN*) modulates neuronal adhesion and migration and is expressed preferentially in the brain [31]. The protein encoded by *FAIM2 *could protect cells from Fas-mediated apoptosis and shows a high level of expression in the hippocampus [32]. It was shown that *OPCML *was expressed predominantly in cerebellum and cerebral cortex [33], whereas *FXYD7 *was expressed preferentially in the brain [34].

The other predicted targets were not previously shown to have brain-specific expression. However, some of these genes, including *GNAO1, SV2A, SYN2, UNC13A *and *NTRK2*, are involved in basic neuron functions (Table 2). Guanine nucleotide binding protein (*GNAO1*) mediates the physiological effects of various neuronal receptors [35]. *SV2A, SYN2 *and *UNC13A *encode proteins which are important for synaptic transmission in the central and peripheral nervous system [36,37]. *NTRK2 *encodes a neurotrophic tyrosine kinase receptor for brain-derived neurotrophic factor (*BDNF*) and is implicated in childhood mood disorder [38]. By contrast, the functions of some high scoring genes in brain remain to be characterized. *HS6ST3 *encodes a Heparan sulphate sulfotransferase which plays a key role in the modulation of fibroblast growth factor signalling [39]. The protein encoded by *SCN2A *forms a voltage-dependent sodium channel and is associated with generalized epilepsy with febrile seizures plus [40]. The corresponding genes of three cDNA sequences (*LOC389073, AA879409 *and *AI186173*) were not determined, and their functions in the brain are not clear. The results suggest that the machine learning approach developed in the present study can be used to identify some interesting targets for further experimental studies.

### Liver-specific gene expression

The liver is a vital organ for human metabolism, and plays key roles in detoxification, plasma protein synthesis, glycogen storage and hormone production. For example, liver is the source and target organ of inflammatory mediators in the pathogenesis of inflammatory response syndrome [41], and it is responsible for the production of coagulation factors. Thus, the liver-specific targets identified in this study might be involved in basic liver functions. We identified 493 liver-specific gene targets with positive scores in the analysis (Additional file 2). The functional analysis of the liver-specific gene targets using RF classifier confirms that many of the predicted targets are enriched for liver-related GO terms (Table 5). For example, the GO terms for inflammatory response contained "acute inflammatory response", "response to wounding" and "activation of plasma proteins involved in acute inflammatory response"; the coagulation-related GO terms included "blood coagulation", "coagulation" and "hemostasis". The expression patterns of the predicted liver-specific genes are visualized with the heat map (Figure 3). Clearly, the transcripts of the predicted targets are predominantly detected in liver samples. Some DNA motifs overrepresented in the promoter regions of predicted liver-specific genes have been identified (Figure 5a), and the candidate transcription factors may be involved in the regulation of the specialized functions of liver (Figure 5b). For example, Chicken ovalbumin upstream promoter (*COUP*) can reduce the expression of hepatocyte nuclear factor 4 (*HNF4*), which plays a key role in blood coagulation and hepatic metabolism [42].

As listed in Table 3, 17 of the top 20 high-scoring genes are involved in the metabolism of human liver. The genes, including *F11, F9, SERPINC1, APOA2, AKR1D1, ACSM2, ITIH2, PON1, CPB2, AFM, NR0B2, ALB, CYP4A11, PGLYRP2 *and *SLC22A7*, have not been annotated as liver-specific genes in UniProt. However, recent studies suggest that some of these genes are expressed preferentially in the liver. For example, *F11, F9 *and *SERPINC1 *are involved in the regulation of blood coagulation cascade [43]. *APOA2 *encodes apolipoprotein which is synthesized mainly in liver and involved in the metabolism of high density lipoprotein [44]. *AKR1D1 *encodes the aldo-keto reductase catalyzing the reduction of steroid hormones [45], whereas *ACSM2 *encodes enzyme catalyzing the activation of medium-chain length fatty acids [46]. The expression and functions of other three predictions (*BG398937, C6 *and *LPA*) have not been documented in the literature.

### Tissue-selective gene prediction

Tissue-specific genes are considered as the special case of tissue-selective genes. Our approach was developed for tissue-specific gene predictions, but its application to tissue-selective gene predictions is straightforward. In this study, the RF classifiers were used to predict the genes that are expressed preferentially in the brain, liver, testis, blood and kidney. The RF classifiers reached high predictive performance for tissue-selective gene prediction (Table 6 and Additional file 3). For example, the classifier for brain-selective gene prediction shows overall accuracy (AC) at 92.70% with Matthews Correlation Coefficient (MCC) = 0.4925. The classifier for liver-selective gene prediction gave predictive performance with the overall accuracy at 96.02% and MCC = 0.7378. It is noteworthy that the classifiers used for tissue-specific gene prediction achieved higher predictive performance than those for tissue-selective gene prediction. For instance, the AUC value of RF classifier for brain-specific gene prediction (AUC = 0.9488, Table 1) is higher than that for brain-selective gene prediction (AUC = 0.9178, Table 6), whereas the RF classifier gave better predictive performance for liver-specific gene prediction (AUC = 0.9777, Table 1) than liver-selective gene prediction (AUC = 0.9547, Table 6). The possible explanation is that the tissue-specific genes are expressed specifically in only one particular tissue type, thus the clear expression patterns of the genes may improve the quality of classifiers and result in high predictive performance for predictions.

**Table 6:** Random Forest classifiers for predicting tissue-selective genes.

Tissue	AC (%)	SN (%)	SP (%)	ST (%)	ROC AUC
Brain	92.70 (± 0.273)	43.55 (± 1.212)	97.60 (± 0.211)	70.58 (± 0.675)	0.9178 (± 0.002)
Liver	96.02 (± 0.341)	65.6 (± 2.499)	99.07 (± 0.191)	82.33 (± 1.293)	0.95467 (± 0.003)
Testis	91.00 (± 0.033)	1.49 (± 0.405)	99.95 (± 0.038)	50.72 (± 0.193)	0.8433 (± 0.004)
Blood	93.29 (± 0.190)	40.20 (± 1.291)	98.53 (± 0.108)	69.37 (± 0.677)	0.9170 (± 0.002)
Kidney	93.62 (± 0.508)	26.43 (± 5.355)	99.73 (± 0.159)	63.08 (± 2.703)	0.9300 (± 0.003)

The values outside and inside brackets are the average value and standard deviation of measures in ten classifier evaluations, respectively.

The RF classifiers gave high predictive performance for predicting genes that have preferential expression in other tissue types. The testis is the male sex gland, which produces sperm, male reproductive cell and sex hormones. The classifier for testis-selective gene prediction reached predictive performance with overall accuracy at 91.00% and ROC AUC = 0.8433. The blood transports oxygen and nutrients to other tissues and carries away waste products from cells. The classifier for blood-selective gene prediction showed overall accuracy at 93.29% with MCC = 0.5109 and ROC AUC = 0.9170. The kidneys play key roles in urinary system. The organs filter waste products from the blood and excrete them in urine. The classifier for kidney-selective gene prediction achieved predictive performance with overall accuracy at 93.62% with MCC = 0.4648 and ROC AUC = 0.9300. The results suggest that our approach can be used to identify the genes that have preferential expression in different types of tissues.

## Conclusions

A machine learning approach has been developed in this study for identifying the human tissue-specific gene targets. Random Forests (RFs) and Support Vector Machines (SVMs) were trained separately with the microarray gene expression data to construct classifiers for prediction. It was shown that the RF classifiers outperform SVM models for tissue-specific gene prediction. 1,408 brain-specific gene targets and 493 liver-specific gene targets were identified using RF classifiers. The predicted targets show clear expression patterns in corresponding tissue samples, and they have functions and regulatory elements consistent with the tissues in GO enrichment analysis and transcription factor prediction. The analysis of high-scoring candidate genes for brain and liver specific expression suggests that our approach can select some interesting targets for further experimental studies. Our approach could also provide useful information for tissue-selective gene prediction. The approach can be used to develop new drug targets for biomedical research and expand our knowledge of tissue-specific expression.

## Competing interests

The authors declare that they have no competing interests.

## Authors' contributions

LW initiated and oversaw the project. ST and LW designed the study. ST conducted the data analysis and drafted the manuscript. JY and LW contributed to result interpretation and manuscript preparation.

## Supplementary Material

Additional file 1 **List of brain-specific gene targets**. The full list of candidate brain-specific genes identified in this study with positive scores. Click here for file

Additional file 2 **List of liver-specific gene targets**. The full list of candidate liver-specific genes identified in this study with positive scores. Click here for file

Additional file 3 **Figure S1 ROC curves to show the RF classifier performances for predicting different tissue-selective genes**. Click here for file
