# Supplementary figures and images for: Genome-wide prediction and analysis of human tissue-selective genes using microarray expression data
Source: BMC Med Genomics. 2013 Jan 23;6(Suppl 1):S10. doi: 10.1186/1755-8794-6-S1-S10 (PMC3552705; doi:10.1186/1755-8794-6-S1-S10)

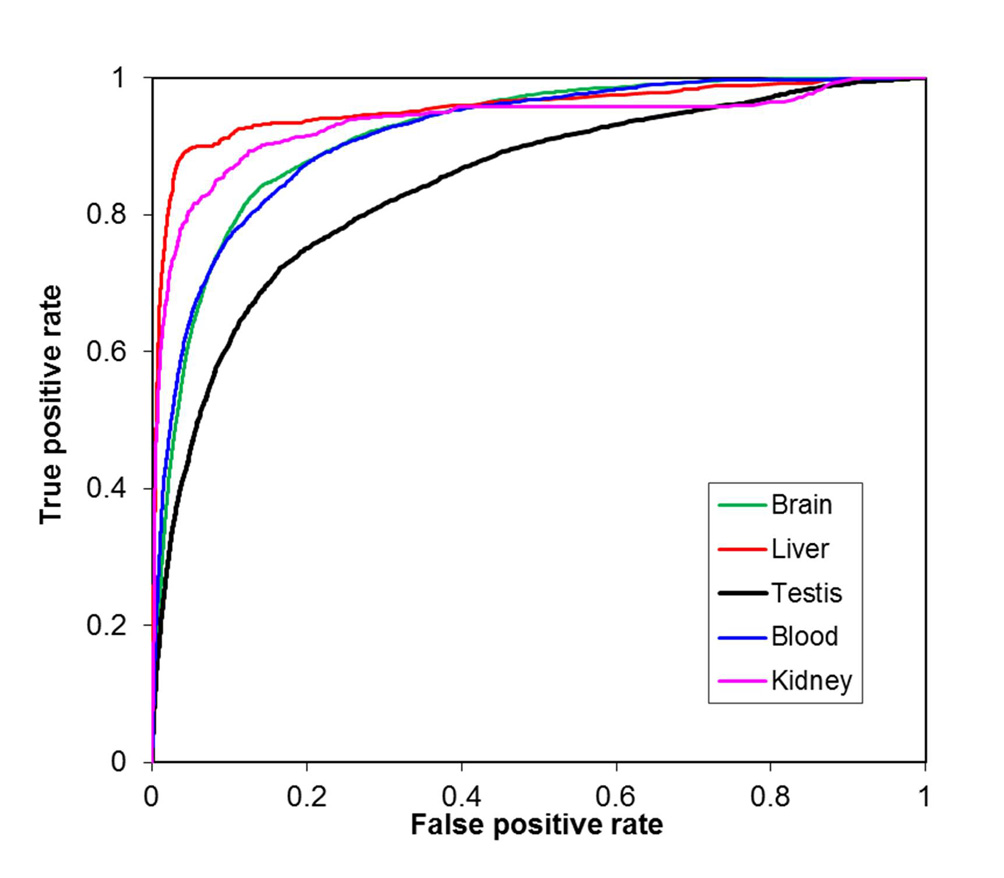

Supplement: Additional file 3 — Figure S1 ROC curves to show the RF classifier performances for predicting different tissue-selective genes. [file 1755-8794-6-S1-S10-S3.jpg]
